# Self-Reported Mental and Physical Measures in Adult Fontan Patients

**DOI:** 10.3390/jcm11143969

**Published:** 2022-07-08

**Authors:** Nili Schamroth Pravda, Oren Zusman, Ilan Richter, Leonard Blieden, Shahar Vig, Ilan Marchushamer, Alexander Dadashev, Yaron Razon, Ran Kornowski, Rafael Hirsch

**Affiliations:** 1 Department of Cardiology, Rabin Medical Center, Petah Tikva 4941492, Israel; nilisc@clalit.org.il (O.Z.); ilanrichtertau@gmail.com (I.R.); lenny.blieden@gmail.com (L.B.); shahar.vig@gmail.com (S.V.); ilanmg@hotmail.com (I.M.); dasahba@clalit.org.il (A.D.); ran.kornowski@gmail.com (R.K.); rhirsch@012.net.il (R.H.); 2 Department of Pediatric Cardiology, Assuta Ashdod Medical Center, Ashdod 7747629, Israel; yrazon@gmail.com

**Keywords:** Fontan, adult congenital heart disease, patient-reported outcomes

## Abstract

Introduction: The Fontan procedure is a palliative operation for patients with single functional ventricles, arising from a heterogeneous group of heart defects. There is a considerable gap in evidence regarding the self-reported physical and mental health of these patients surviving to adulthood. Methods and Results: We administered the PROMIS^®^ Global Short Form (v 1.2) to Fontan patients during their scheduled clinic visits during 2017–2018. The raw PROMIS scores were subsequently converted to standardized T-scores, where the mean performance was 50 for the general population. We used Cronbach’s alpha to assess reliability, with >0.8 considered good. A total of 42 patients were included. The median age was 30 (IQR: 24–34) years and 59% (95% CI: 43–74%) were female. The median time from birth to operation was 4.5 (IQR: 3–8) years, with 55% having an extracardiac Fontan. The questionnaire had good internal reliability with an alpha of 0.87. Seventy-one percent of respondents rated their overall health as “excellent” or “good”. The mean T-score for physical health was 46.6, lower than the age-group mean (51.6, *p* < 0.001). The mean T-score for mental health was 53.3, higher than the age-group mean (48.5, *p* < 0.001). T-scores showed strong correlation with each other (r = 0.7) and weak correlation with age and time from procedure. There was no association of T-score with diagnosis or operation type. Conclusions: Adult Fontan patients report better mental health despite worse reporting physical health compared with the age group means. Patient-reported measures can provide clinically meaningful insights about the care of patients with complex congenital heart disease.

## 1. Introduction

The Fontan palliation, first described for tricuspid atresia, has allowed for the survival of many patients with complex congenital heart lesions into adulthood [1]. This palliation is designed to provide blood flow in series to the pulmonary and systemic circulations in patients with a single functional ventricle. A series of operations form the Fontan circulation, which allows systemic venous blood to flow directly to the pulmonary circulation. As the majority of these patients survive into adulthood, the incorporation of quality-of-life assessments into patient management is pertinent [2].

For many years, decisions in clinical care and outcomes have been subject to clinicians’ objective assessments of patients’ health. However, subjective measures of an individual patient’s wellbeing and perceived effects of treatments should play a key role in the clinical management of patients. A patient-reported outcome measure (PROM) is a measure of a patient’s health status directly reported by the patients, and can provide useful insights into self-perceived wellbeing secondary to a diagnosis and/or treatment, without interpretation of the patient’s response by a clinician [3].

## 2. Methods

We administered the PROMIS^®^ (*Patient-Reported Outcomes Measurement Information System*) Global Short Form (v 1.2) to Fontan patients during scheduled ACHD clinic visits during 2017–2018 (Supplementary Table S1). This form consists of ten global health items that represent five core PROMIS domains (physical function, pain, fatigue, emotional distress, and social health). Four items measure physical health (questions 3, 7, 9, and 10) and four items measure mental health (questions 2, 4, 5, and 8). Each item was graded according to patient satisfaction. We also collected relevant demographic and medical data from patient records. Subsequently, raw PROMIS scores were standardized as T-scores using Health Measures Scoring Services, such that the general population mean score was 50 points [4]. We used Cronbach’s alpha to assess PROMIS score reliability, using a threshold of 0.8 for good reliability. This research project was approved by the Local Helsinki Ethics Committee. A total of 43 patients were approached for participation, of which 42 patients completed the questionnaire.

## 3. Results

A total of 42 patients were included. Baseline characteristics are shown in Table 1. The median age was 30 (IQR: 24–34) years and 59% (95% CI: 43–74%) were female. The median time from birth to operation was 4.5 (IQR: 3–8) years. All patients had primary complex congenital heart disease necessitating a Fontan procedure. The primary pathology was tricuspid atresia in 17 patients (40.5%), double inlet left ventricle in 14 patients (33%), 4 patients with atrioventricular septal defect (9.5%), and 4 with transposition of the great vessels associated with other complex lesions (9.5%). In total, 55% had an extracardiac Fontan.

## 4. Objective Measures

The mean follow-up from time of Fontan procedure was 22 (95% CI: 20–25) years. During this time, six patients underwent re-operation and four patients underwent fenestration of the Fontan. Previous morbidity among these patients included 11 patients with symptomatic arrythmias, 2 patients with cirrhosis, 2 patients with episodes of hemoptysis, and 4 events of cerebrovascular accidents (CVA).

## 5. Patient-Reported Outcomes

**Figure 1:**
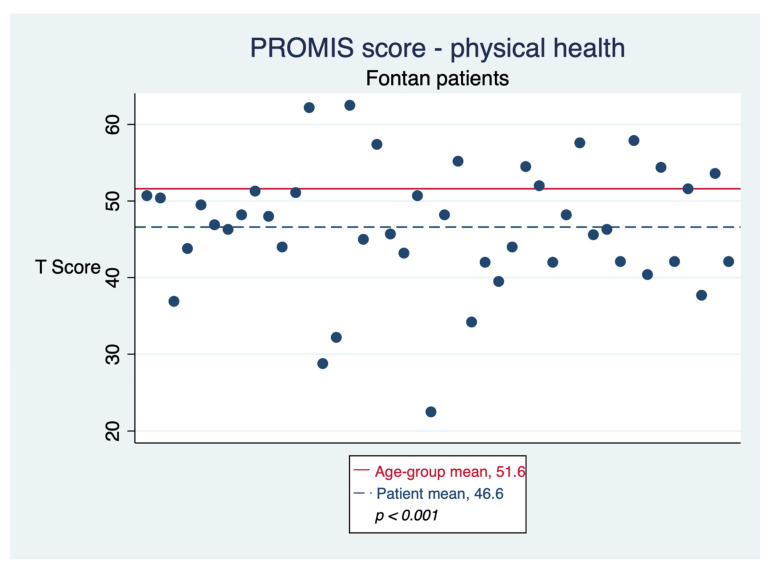
Physical health as reported by Fontan patients.

**Figure 2:**
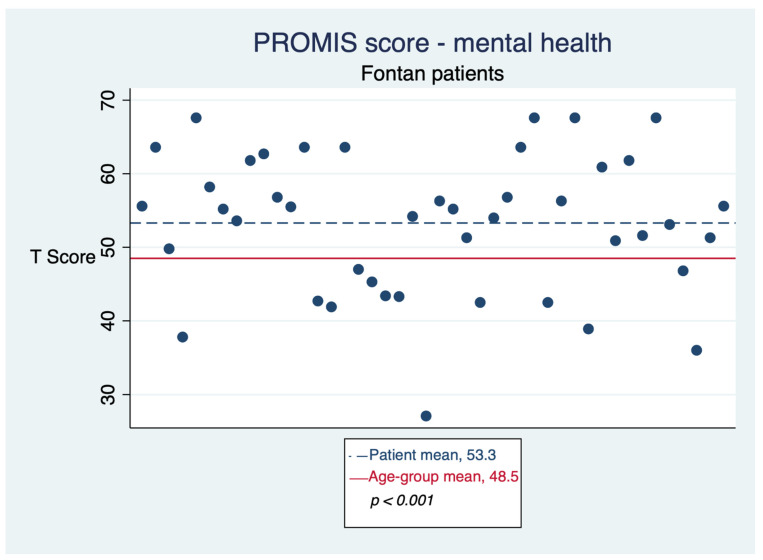
Mental health as reported by Fontan patients.

The questionnaire showed good internal reliability, with an alpha of 0.87. Seventy-one percent of respondents rated their overall health as “excellent” or “good”. The mean T-score for physical health was 46.6 (95% CI: 44.1–49.1), lower than the age-group mean (51.6, *p* < 0.001), as seen in Figure 1. The T-score for mental health was 53.3 (95% CI: 50.3–56.1), higher than age-group mean (48.5, *p* < 0.001), as seen in Figure 2. T-scores showed strong correlation with each other (r = 0.7) and weak correlation with age and time to procedure. No association between T-score and diagnosis or operation type was found. T-scores did not differ by gender (mean T-score for mental health, 54.4 for males vs. 52.3 for females, *p* = 0.46; mean T-score for physical health, 48.8 for males vs. 45.0 for females, *p* = 0.14) or by having developed any complication (tachyarrhythmias, cirrhosis, hemoptysis, stroke, or venous thromboembolism) (mean T-score for mental health, 54.0 for patients with any complication vs. 52.7 for those without, *p* = 0.66; mean T-score for physical health, 45.5 for patients with any complication vs. 47.2 for those without, *p* = 0.52).

## 6. Discussion

Our study shows that the self-reported overall health of adult patients who have undergone the Fontan operation is good or excellent. While the self-reported physical health of the cohort was lower than age-group mean, mental health was reported as better.

There is an increasing awareness of the importance and value of PRO measures in cardiovascular disease, as measures that aim to exclude physician bias and act as a tool to evaluate management options and quality-of-life outcomes [5]. These measures are an important assessment tool that can help provide holistic patient-centered management to these patients.

The Fontan circulation is a delicate circulation characterized by chronically elevated systemic venous pressures, altered pulmonary hemodynamics, and preload deprived ventricle. This circulation is associated with unique long-term complications. These patients are regarded as having a severe congenital heart defect. They require long-term follow-up and are often on chronic medication [6]. Considering this, the finding that these patients have lower self-reported physical health than the general population is not surprising.

Encouragingly, the self-reported mental health of adult Fontan patients was better than the age-group mean. While an elucidation of this finding must be multifactorial in nature, one explanation could be that these patients have experienced multiple surgeries and hospitalizations, resulting in resiliency and a heightened appreciation for their health.

While it is encouraging that patients with Fontan circulation report mental wellbeing above that of the general population, physicians should be aware of the discrepancy between the mental wellbeing and the objective health status of these patients. Previous reports have shown that patients with congenital heart disease over-estimate their functioning when compared with objective measures [7]. Gratz et al. found similar results in a mixed cohort of patients with congenital heart disease, who reported excellent quality of life. This was despite the fact that most patients had considerable exercise capacity limitations. Gratz et al. concluded that self-estimated physical functioning poorly predicted exercise capacity in those with congenital heart disease [8]. This further serves to highlight the importance of obtaining objective measures of health, through the use of imaging/physiological modalities, to assess the physical status of these patients. These modalities can help identify the functional limitations of the patients and highlight subtle changes in physical health in order to address them early.

The literature on PROMs in adult Fontan patients is scarce. Overgraad et al. performed a case-control study comparing PROMs in 62 patients with single ventricle physiology that survived to adulthood with healthy controls [9]. They found that patients had good functional status and were able to lead a normal life with full-time work or study. However, even those in good functional status had poorer general health and physical functioning than healthy controls.

Our study adds contemporary data to the growing body of literature on adult survivors of functionally single ventricle conditions corrected with a Fontan circulation. The strength of our data is the relatively large number of patients seen at a single center. Most of our patients have been followed up at our center since childhood and have had a prolonged, steady relationship with our adult congenital heart team. This element of stability and continuity of care could have had a reinforcing influence on our patients.

Our study has some inherent limitations. We report from a single center and our results may not be generalized to other centers. Our cohort is of adult patients with Fontan circulation and there is inherent survival bias. Our cohort was relatively young, with a mean age of 30 years, and we may expect to encounter more Fontan-related complications at a later age. This may have affected the perceived health of the cohort, although the use of an age-group mean for comparison minimizes this bias. Furthermore, there is also an element of reporting bias owing to the nature of PROMs. We did not assess outcomes as per type of Fontan because of the small numbers of patients in each category.

In conclusion, although adult patients after the Fontan operation rate their physical health lower than the general population, their perception of their mental health is better than that of their healthy peers.

## Figures and Tables

**Table 1:** Characteristics of the cohort.

Number of patients	n = 42
Age (years)	30 (IQR: 24–34)
Female (%)	25 (59)
Median time from birth to Fontan (years)	4.5 (IQR: 3–8)
Primary Pathology	
Tricuspid atresia (%)	17 (40.5)
Double inlet left ventricle (%)	14 (33)
Atrioventricular septal defect	4 (9.5)
Transposition of the great vessels associated with other complex lesions (%)	4 (9.5)
Extracardiac Fontan (%)	55
Mean follow-up from time of Fontan (years)	22 (95% CI: 20–25)
Re-operation during follow up (%)	6 (14)
Fenestration of the Fontan	4 (9.5)
Symptomatic arrythmia (%)	11 (26)
Cirrhosis (%)	2 (5)
Hemoptysis (%)	2 (5)
Cerebrovascular accident (%)	4 (9.5)

## Data Availability

Data from Department of Cardiology, Rabin Medical Center.

## Supplementary Materials

The following supporting information can be downloaded at https://www.mdpi.com/article/10.3390/jcm11143969/s1, Table S1. PROMIS global short form (V1.2).
