# Supplementary material for: Self-Reported Mental and Physical Measures in Adult Fontan Patients
Source: J Clin Med. 2022 Jul 8;11(14):3969. doi: 10.3390/jcm11143969 (PMC9323562; doi:10.3390/jcm11143969)
Supplement: Supplementary file 1 [file jcm-11-03969-s001.zip › jcm-1769916-supplementary.pdf]

Table S1. PROMIS global short form (V1.2)

		Excellent	Very good	Good	Fair	Poor
1	In general, would you say your health is….					
2	In general, would you say your quality of health is….					
3	In general, how would you rate your physical health?					
4	In general, how would you rate your mental health?					
5	In general, how would you rate your satisfaction with your social activities and relationships?					
6	In general, please rate how well you carry our your usual social activies and roles. (This includes activities at home, at work, and in your community, and responsibilities as a parent, child, spouse, employee, friend etc)					
		Completely	Mostly	Moderately	A little	Not at all
7	To what extent are you able to carry out your everyday physical activities such as walking, climbing stairs, carrying groceries, or moving a chair?					
**In the past 7 days**						
		Never	Rarely	Sometimes	Often	Always
8	How often have you been bothered by emotional problems such as feeling anxious, depressed, or irritable?					
		None	Mild	Moderate	Severe	Very severe
9	How would you rate your fatigue on average?					
10	How would you rate your pain on average?	0=No Pain------------------------------→10= worst imaginable pain				
